# PDGF activation in PGDS-positive arachnoid cells induces meningioma formation in mice promoting tumor progression in combination with *Nf2* and *Cdkn2ab* loss

**DOI:** 10.18632/oncotarget.5296

**Published:** 2015-09-24

**Authors:** Matthieu Peyre, Céline Salaud, Estelle Clermont-Taranchon, Michiko Niwa-Kawakita, Stephane Goutagny, Christian Mawrin, Marco Giovannini, Michel Kalamarides

**Affiliations:** ^1^ Department of Neurosurgery, AP-HP, Hôpital Pitié-Salpêtrière, Paris, France; ^2^ Université Paris 6 - Pierre et Marie Curie, Paris, France; ^3^ CRICM INSERM U1127 CNRS UMR 7225, Institut du Cerveau et de la Moelle Epinière, Paris, France; ^4^ Inserm U944, CNRS U7212, Université Paris VII, Institut Universitaire d'Hématologie, Paris, France; ^5^ Department of Neurosurgery, AP-HP, Hôpital Beaujon, Clichy, France; ^6^ Department of Neuropathology, Otto-von-Guericke Universität, Magdeburg, Germany; ^7^ Department of Head and Neck Surgery, David Geffen School of Medicine, University of California Los Angeles, Los Angeles, CA, USA

**Keywords:** meningioma, PDGF, Nf2, mouse model, RCAS

## Abstract

The role of PDGF-B and its receptor in meningeal tumorigenesis is not clear. We investigated the role of PDGF-B in mouse meningioma development by generating autocrine stimulation of the arachnoid through the platelet-derived growth factor receptor (PDGFR) using the RCAStv-a system. To specifically target arachnoid cells, the cells of origin of meningioma, we generated the *PGDStv-a* mouse (Prostaglandin D synthase). Forced expression of PDGF-B in arachnoid cells *in vivo* induced the formation of Grade I meningiomas in 27% of mice by 8 months of age. *In vitro*, PDGF-B overexpression in PGDS-positive arachnoid cells lead to increased proliferation.

We found a correlation of PDGFR-B expression and *NF2* inactivation in a cohort of human meningiomas, and we showed that, in mice, *Nf2* loss and PDGF over-expression in arachnoid cells induced meningioma malignant transformation, with 40% of Grade II meningiomas. In these mice, additional loss of *Cdkn2ab* resulted in a higher incidence of malignant meningiomas with 60% of Grade II and 30% of Grade III meningiomas. These data suggest that chronic autocrine PDGF signaling can promote proliferation of arachnoid cells and is potentially sufficient to induce meningiomagenesis. Loss of *Nf2* and *Cdkn2ab* have synergistic effects with PDGF-B overexpression promoting meningioma malignant transformation.

## INTRODUCTION

Meningiomas account for approximately one-third of all primary central nervous system tumors and are the most common brain tumor in adults over 35 years of age [1]. Most meningiomas are benign and do not recur after complete surgical resection, resulting in prolonged disease free survival and low morbidity. In contrast, a subset of recurrent and/or histologically aggressive meningiomas (15–25% of tumors, WHO Grade II and III) presents with high morbidity and mortality [2–4], and repeated surgeries, radiosurgery/radiotherapy are the only option. Despite recent advances in understanding molecular mechanisms of meningioma development [5, 6], there is no available drug treatment.

We have demonstrated that Nf2 loss in arachnoid cells is sufficient to initiate meningioma development in mice [7], and identified a PGDS(Prostaglandin D2 synthase)-positive arachnoid cell as the cell of origin of meningioma [8]. Moreover, as in humans, *Nf2* and *Cdkn2ab* loss cooperate to promote progression to histologically aggressive meningiomas [9, 10].

In human meningioma, there is accumulating evidence that platelet-derived growth factor (PDGF) stimulates tumorigenesis. PDGF ligands AA and BB and PDGF receptor-B (PDGFR-B) have been detected by immunohistochemistry and Western Blot in the majority of meningiomas of all grades [11–14]. Early studies have also shown that normal arachnoid cells express PDGF receptors, almost exclusively of the β-type [14, 15]. Administration of PDGF-B to meningioma cells in culture stimulates growth and activates mitogen-activated protein kinases and c-fos [16]. However, the exact importance of PDGF-B / PDGFR-B signaling in meningiomas remains elusive, especially in relation with *NF2* inactivation.

To specifically overexpress PDGF-B in arachnoidal cells, we used the RCAS/tv-a system in which oncogene carrying RCAS (replication-competent avian sarcoma-leukosis virus long terminal repeat with splice acceptor) retroviruses infect specific somatic cells carrying the tv-a receptor in tv-a transgenic mice [17]. By generating transgenic mice expressing tv-a under the control of tissue specific promoters, exclusive infection by RCAS has been obtained to tv-a-expressing cells, allowing the generation of several mouse glioma models, including PDGF-B-driven tumors [18, 19] and demonstrating that abnormal PDGF signaling can contribute to the etiology of brain tumors. In order to specifically target arachnoid cells, we chose the *PGDS* promoter to generate a new transgenic *PGDStv-a* strain. Here we show that PDGF-B overexpression in arachnoid mouse cells induces the development of benign meningiomas synergizing with *Nf2* and *Cdkn2ab* loss for malignant histological progression.

## RESULTS

### Expression of PDGFR-B is correlated with *NF2* status in human meningiomas

**Figure 1:**
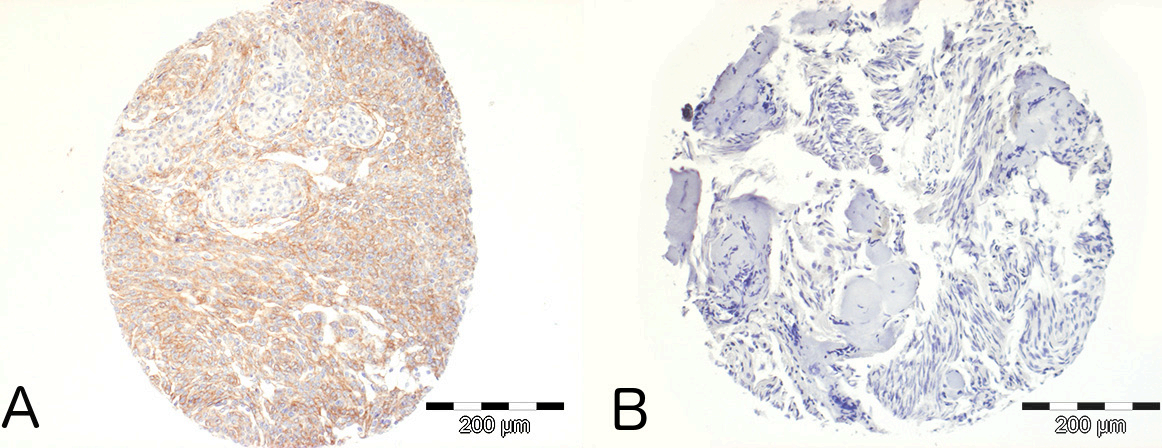
Immunohistochemical analysis of PDGFR-B expression in human meningiomas **A.** Example of a *NF2*- Grade II meningioma demonstrating strong positivity. **B.** Example of a *NF2*+ Grade I meningioma demonstrating no immunopositivity.

PDGF-B and PDGFR-B are widely expressed in human meningiomas [11]. To assess the relationship between PDGF-B expression and *NF2* status, we performed PDGFR-B immunohistochemistry on a Tissue Array of 54 meningiomas of all grades with known *NF2* status. We analyzed 36 *NF2*- meningiomas (defined as presenting either an identified *NF2* mutation and 22q LOH, or 22q LOH with no NF2 protein expression on Western blot) and 18 *NF2*+ meningiomas (defined as presenting NF2 protein expression with no 22q LOH and no *NF2* mutation). PDGFR-B immunopositivity was found in 54% of meningiomas and statistically more frequent in NF2- tumors compared to *NF2*+ tumors (23/36 vs. 6/18, χ^2^, *p* = 0,03, Figure 1). No correlation between PDGFR-B immunopositivity and meningioma histological grade was found.

### Generation and functional testing of the *PGDStv-a* mouse

To overexpress PDGF-B in arachnoid cells *in vivo*, we have generated a new transgenic mouse strain where the *tv-a* receptor is expressed under the control of the endogenous PDGS gene promoter using a knock-in approach [8]. A nuclear-targeted *tv-a* receptor gene was integrated into the PGDS coding region by replacing exon 1 of the murine *PGDS* gene (Figure S1, supplementary data).

**Figure 2:**
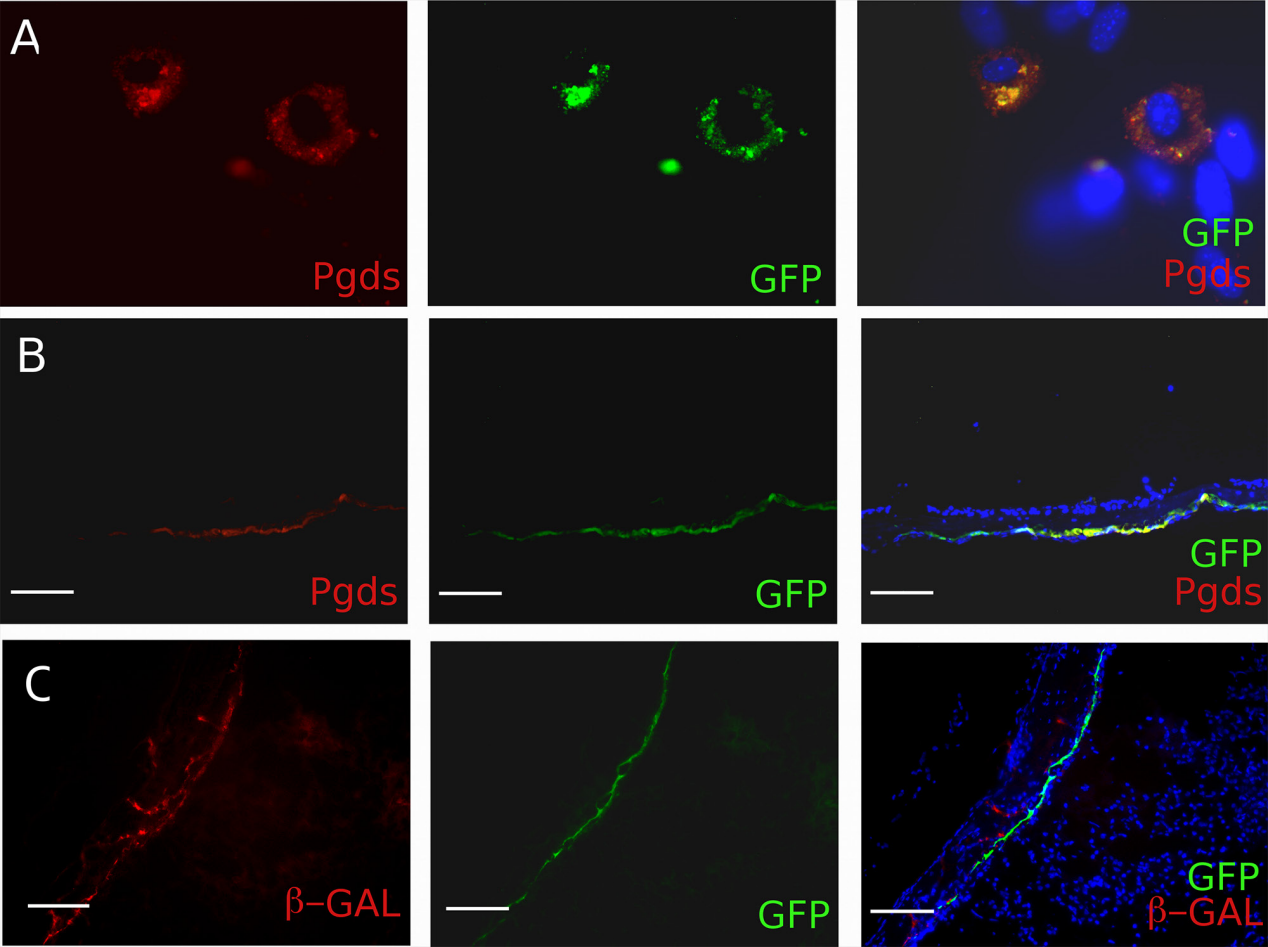
*In vitro* and *in vivo* characterization of the PDGDStv-a mouse **A.** Functional analyses of the *PGDStv-a* transgenic construct in a culture of arachnoidal cells. Infection with RCAS-eGFP was directed to cells with arachnoid morphology and the enhanced green fluorescent protein (eGFP)-infected cells co-expressed PGDS (red cytoplasmic staining). **B.** *In vivo* characterization of PN7 *PGDStv-a* mouse brains and meninges. Arachnoid PGDS positive cells forming a thin layer were found to be positive for GFP after transfection with RCAS-eGFP. **C.** Co-localisation of X-gal (red staining) and GFP stainings in PGDS positive arachnoid cells demonstrating the possibility to co-transfect the same cells with both RCAS and Adenovirus vectors.

To evaluate the *in vitro* expression of the transgene, we established primary arachnoid cell cultures from neonatal *PGDStv-a* brains and infected them with supernatant from DF-1 cells producing RCAS-eGFP (Figure 2A). Approximately 5–10% of the cells were infected and were large in size with oval nuclei, reminiscent of arachnoid cap cells. Infected cells were analyzed with immunocytochemistry and identified by GFP immunopositivity. As expected, GFP-positive cells had a strong PGDS immunopositivity (Figure 2A).

To confirm that the transgene was functionally expressed *in vivo*, we injected DF-1 cells producing RCAS-eGFP retrovirus in the sub-dural space of newborn mice at PN1 and analyzed PN5 meninges. The GFP-positive infected arachnoid mouse cells simultaneously expressed PGDS and formed a delicate layer surrounding the brain, thus perfectly adopting the shape of the arachnoid layer (Figure 2B). As we aimed at deleting *Nf2* using Cre-loxP system and activating PDGF-B using the RCAS-/tv-a system in the same cell, we injected newborn mice in the sub-dural space with DF-1 cells producing RCAS-eGFP retrovirus to test the RCAS/tv-a recombination and Adenovirus Ad*β-Gal* to test the Cre-loxP recombination. We then analyzed the arachnoid layer and demonstrated co-infection of meningioma progenitor cells with both RCAS virus and Adenovirus (Figure 2C).

In conclusion, we found that in the *PGDStv-a* mouse functional tv-a expression is restricted to PGDS positive cells and that both *in vitro* and *in vivo* retroviral infections resulted in specific transgene expression, alone or in combination with adenoviral infection.

### Effect of overexpression of the PDGF-B oncogene in PGDS-positive arachnoid cells alone or in combination with *Nf2* inactivation

**Figure 3:**
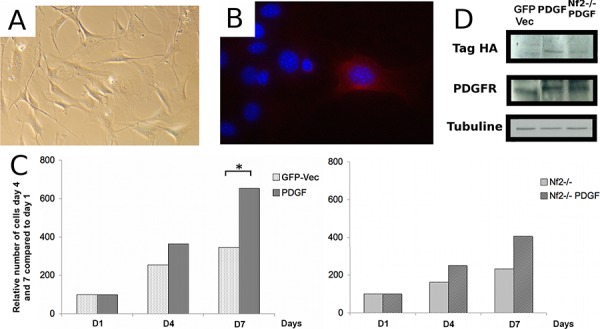
Effect of oncogenic stimulation by platelet-derived growth factor B (PDGF-B) on proliferation of arachnoidal cells in culture alone or in combination with Nf2 inactivation **A.** Morphology of arachnoid cells in culture. **B.** Hemagglutinin A (HA)-immunocytochemistry in arachnoid cells *in vitro*. **C.** Effect of PDGF-B overexpression on the growth of arachnoid cells *in vitro*, alone (left) or in combination with Nf2 bi-allelic inactivation (right) **D.** Western blot analysis of arachnoid cells demonstrating increased PDGFR immunoreactivity in arachnoidal cells infected with RCAS-PDGF-B compared to controls.

To analyze the effect of overexpression of PDGF-B *in vitro*, we cultured arachnoid cells taken from the meninges of the skull vault of *PGDStv-a;Nf2*^*flox2*/flox2^ mice. Cells in culture displayed an enlarged and flattened phenotype as previously reported in human meningioma cell cultures [20] (Figure 3A). Next, we co-infected cells with RCAS-PDGF-B and *AdCre*. Viral infection was verified by HA (Human influenza hemagglutinin)-tag immunostaining of infected cells (Figure 3B). Because of the rapid senescence of arachnoid cells in culture, experiments were performed using cells at early passages. For the analysis, the number of cells at days 4 and 7 after infection was determined and calculated as fold change of cell number compared to day 1 (Figure 3C). Overexpression of PDGF-B significantly increased the proliferation of arachnoid cells compared to the control cells transfected with RCAS-GFP (Student *t*-test, *p* < 0.05). Similarly, concomitant overexpression of PDGF-B and *Nf2* inactivation increased the proliferation of arachnoid cells compared to cells with *Nf2* inactivation alone. In human meningioma primary cultures overexpression of PDGF-B increases the number of PDGF-B receptors (PDGFR-B) by an autocrine loop [16, 21]. Likewise, in our system, overexpression of PDGF-B by RCAS infection increases the expression of PDGFR-B in cultured mouse arachnoid cells (Figure 3D).

### PDGF-B overexpression alone results in meningioma formation while promoting malignant progression in a null *Nf2* and *Cdkn2ab* background

To investigate the potential role of PDGF-B in meningioma initiation, we generated a cohort of 26 *PGDStv-a*;RCAS-PDGF-B mice and 19 *PGDStv-a*;RCAS-X control mice, injected with an empty RCAS vector (RCAS-X). After a mean follow-up of 8.0 months, we found 27% of benign meningiomas, mostly located at the convexity, and 33% of meningothelial proliferations in *PGDStv-a*;RCAS-PDGF-B mice (Table 1). No meningeal lesions were found in the control *PGDStv-a*;RCAS-X cohort after a mean follow-up of 9.0 months. Interestingly, five PDGF-B-injected mice developed hydrocephalus before 3 months of age associated with meningothelial proliferations and/or meningiomas.

**Table 1:** Summary of the phenotypic consequences of PDGF-B overexpression in mouse PGDS+ arachnoidal cells *in vivo*, alone or in combination with *Nf2* and *Cdkn2ab* inactivation

	*PGDStv-a*; PDGF-B (*n* = 26)	*PGDStv-a*; PDGF-B; AdCre; *Nf2*^flox/flox^ (*n* = 29)	Control series AdCre; *Nf2*^flox/flox^ (*n* = 25)	*PGDStv-a*; PDGF-B; AdCre; *Nf2*^flox/flox^; *Cdkn2ab*^−/−^ (*n* = 19)	Control series AdCre;*Nf2*^flox/flox^; *Cdkn2ab*^−/−^ (*n* = 53)
*Mean survival (months)*	*8.0*	*6.3*	*6.4*	*1.8*	*3.5*
Meningiomas	7 (27%)	15 (52%)	4 (16%)	15 (79%)	38 (72%)
-convexity	7	14	0	11	18
-skull base	0	1	4	3	20
Meningothelial proliferation	8 (33%)	11 (38%)	8 (32%)	8 (42%)	5 (9%)
Glioma	23 (88%)	14 (48%)	0	15 (79%)	0
Hydrocephalus	17 (32%)	2 (7%)	1 (3%)	0	17 (32%)

The results of the AdCre; *Nf2*^flox/flox^; *Cdkn2ab*^−/−^ control series were taken from Peyre et al. (9)

**Figure 4:**
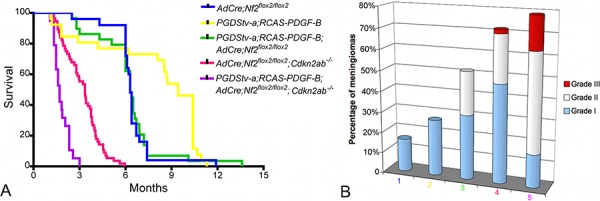
Role of PDGF-B alone or in association with Nf2 loss and Cdkn2ab loss in meningiomagenesis **A.** Survival curves of *PGDStv-a*;RCAS-PDGF-B mice (*n* = 26), *AdCre*;*Nf2*^*flox2*/flox2^ mice (*n* = 25), *PGDStv-a* het; RCAS-PDGF-B; *AdCre*;*Nf2*^*flox2*/flox2^ mice (*n* = 29), *AdCre*;*Nf2*^*flox2*/flox2^; *Cdkn2ab*^−/−^ mice (*n* = 53) and *PGDStv-a*;RCAS-PDGF-B;*AdCre*;*Nf2*^flox2/flox2^;*Cdkn2ab*^−/−^ (*n* = 19) mice after perinatal injection. **B.** Proportion of meningioma histological grades in the different mice cohorts : **1.** *PGDStv-a*;RCAS-PDGF-B mice, **2.** *AdCre*;*Nf2*^flox2/flox2^ mice, **3.** *PGDStv-a*;RCAS-PDGF-B;*AdCre*;*Nf2*^flox2/flox2^ mice, **4.** *AdCre*;*Nf2*^*flox2*/flox2^;*Cdkn2ab*^−/−^ mice, **5.** *PGDStv-a*;RCAS-PDGF-B;*AdCre*;*Nf2*^flox2/flox2^;*Cdkn2ab*^−/−^ mice.

**Figure 5:**
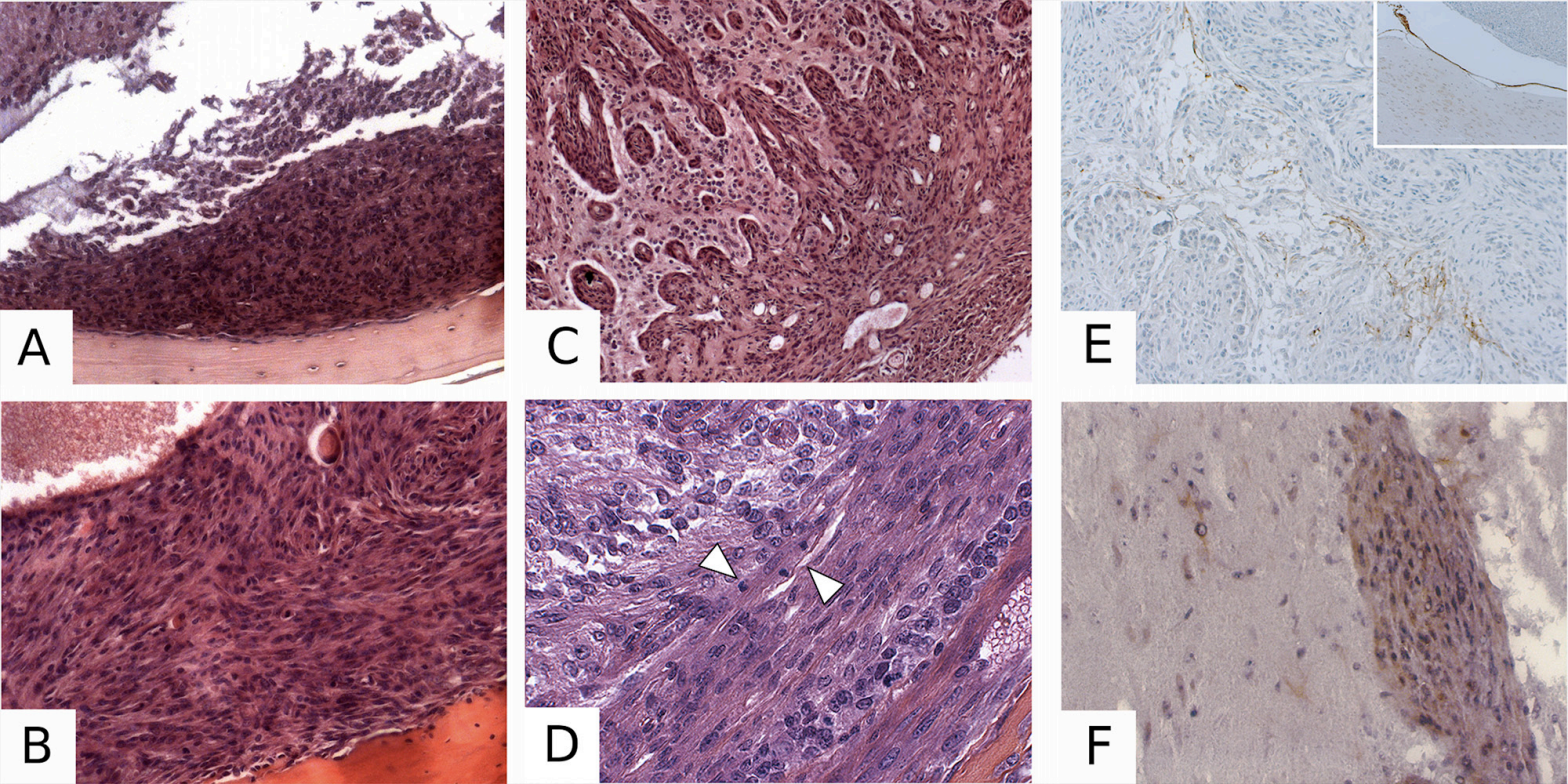
Pathological characterization of PDGF-induced meningiomas **A.** H&E-stained section illustrating a Grade I meningioma in a *PGDStv-a*;RCAS-PDGF-B mouse. **B.** Example of a Grade II meningioma in a *PGDStv-a*;RCAS-PDGF-B;*AdCre*;*Nf2*^flox2/flox2^ mouse. **C.** Grade III mouse meningioma with brain invasion in a *PGDStv-a*;RCAS-PDGF-B;*AdCre*;*Nf2^flox2^*/flox2;*Cdkn2ab*^−/−^ mouse. **D.** Grade III mouse meningioma with a high mitosis count in a *PGDStv-a*;RCAS-PDGF-B;*AdCre*;*Nf2*^*flox2*/flox2^; *Cdkn2ab*^−/−^ mouse. Arrows indicate two mitoses in a single 63x power field. Example of a Grade III meningioma with focal PGDS positive immunostaining **E.** with the surrounding arachnoid layer as a positive control (insert). HA immunostaining showing positive **F.** meningioma cells infiltrating the brain. Some of oligodendroglial cells are also HA-positive.

To determine if *Nf2* loss alone or in combination with *Cdkn2ab* loss could promote progression of PDGF-B induced benign meningiomas, we generated cohorts of *PGDStv-a*;RCAS-PDGF-B;*AdCre;Nf2*^*flox2*/flox2^ and *PGDStv-a*;RCAS-PDGF-B*;AdCre;Nf2*^*flox2*/flox2^; Cdkn2ab^−/−^ mice. In *PGDStv-a*;RCAS-PDGF-B;*AdCre;Nf2*^*flox2*/flox2^mice the rate of meningioma development was significantly higher (15 of 29 mice; 52%) compared to *AdCre;Nf2*^*flox2*/flox2^ mice (4 of 25 age-paired mice; 16%; χ^2^, *p* = 0,02) (Table 1). In *PGDStv-a*;RCAS-PDGF-B*;AdCre;Nf2^flox2/flox2^*; Cdkn2ab*^−/−^* mice, the frequency of meningiomas (15 of 19 mice; 79%) was not statistically different compared to control *AdCre;Nf2^flox2/flox2^*; Cdkn2ab*^−/−^* mice (38 of 53 mice; 72%). To evaluate the role of PDGF-B activation in meningioma malignant progression, we classified the mouse meningiomas according to the GEM meningioma pathological classification (Figure 4) [9]. We observed 60% (9/15) of grade I and 40% (6/15) grade II meningiomas in *PGDStv-a*;RCAS-PDGF-B;*AdCre;Nf2^flox2/flox2^* mice (Figure 5A). Of the six grade II meningiomas, four presented at least one mitosis and two showed subpial invasion (Figure 5B). In the group of *PGDStv-a*;RCAS-PDGF-B*;AdCre;Nf2^flox2/flox^*;Cdkn2ab*^−/−^* mice, we observed 33% (5/15) of grade I meningiomas, 47% (7/15) of grade II meningiomas and 20% (3/15) of grade III meningiomas. Of the seven grade II meningiomas, one tumor showed subpial invasion and six had at least one mitosis. Among the 3 grade III meningiomas, two presented an architectural dedifferentiation with sarcoma-like appearance and one preserved a meningothelial differentiation but with more than 10 mitosis per field (Figure 5C, 5D). The rate of histologically aggressive meningiomas (grades II and III) was statistically higher in *PGDStv-a*;RCAS-PDGF-B*;AdCre;Nf2^flox2/flox2^*;Cdkn2ab*^−/−^* mice compared to *AdCre;Nf2^flox2/flox2^*;Cdkn2ab*^−/−^* mice (χ^2^, *p* = 0,002 and *p* = 0.03). As for human aggressive meningiomas, brain invasion by direct breaching of pial surface and/or along Virchow-Robin spaces was observed in grades II and III mouse meningiomas

To demonstrate that meningiomas were induced by RCAS-PDGF-B infection, we performed HA-tag immunohistochemistry in 7 selected meningiomas of all grades. Immunopositivity was found in 50–90% of labeled cells within the tumors (Figure 5F). PGDS immunohistochemistry revealed some cases of strong PGDS immunostaining within specific regions of the tumor (Figure 5E).

In addition to meningiomas, we also found several gliomas in all the *PGDStv-a* cohorts (Table 1). *PGDStv-a*;RCAS-PDGF-B mice developed oligodendroglioma while in *PGDStv-a*; RCAS-PDGF-B*;AdCre;Nf2^flox2/flox2^* mice and *PGDStv-a*;RCAS-PDGF-B*;AdCre; Nf2^flox2/flox2^*;Cdkn2ab*^−/−^* mice, all tumors grades were found, from WHO Grade II gliomas to WHO Grade IV glioblastomas. Most tumors resembled oligodendroglioma, but some had mixed oligodendrocytic and astrocytic components as shown by GFAP immunopositivity (data not shown), as previously described in models of PDGF-driven gliomas derived from oligodendroglial progenitors [19]. In conclusion, we demonstrated that *in vivo* PDGF-B overexpression in mouse arachnoidal cells promotes meningioma initiation and cooperates with *Nf2* and *Cdkn2ab* loss to promote histological meningioma progression.

## DISCUSSION

This study explores the role of PDGF-B in meningioma tumorigenesis using an innovative approach combining conditional mutagenesis and the RCAS/tv-a system to recapitulate the genetic alterations found in human meningioma progression. This is the first case of combination of Cre-loxP and RCAS-tv-a systems using two different types of viral vectors (adenovirus and retrovirus). Seidler et al. [22] generated a mouse model for Cre-inducible tv-a expression by insertion of a silenced tv-a/lacZ cassette into the ubiquitously expressed *Rosa26* locus. Others [23, 24] combined conditional gene knockout and oncogene overexpression in the same tv-a expressing cell type by simultaneously injecting two RCAS vectors with the Cre recombinase and the oncogene of interest. Use of two RCAS viruses in our model would have hampered a direct comparison with our previous models, due to the change in the method of delivery of the Cre recombinase to arachnoid cells.

We showed that PDGF overexpression alone results in meningioma formation *in vivo*, the relatively short latency of meningioma development in these mice arguing against the need for additional mutations. A link between PDGFR expression and NF2 loss has been demonstrated in human schwannoma [25]. Merlin-deficient schwannoma cells show accumulation of growth factors receptors at the plasma membrane, including PDGFR-B [26], with high levels of receptors associated with an increase of phosphorylated receptors. These molecular phenotypes were correlated with the expression of merlin: its reintroduction into *Nf2*^−/−^ Schwann cells markedly reduced the levels of PDGFR-B. Moreover, in a schwannoma cell line, merlin was also shown to decrease membrane levels of PDGFR-B by inducing its degradation [27]. A statistically higher expression of PDGFR-A has been found in meningiomas with monosomy 22 compared to meningiomas with normal karyotype, while PDGFR-B was ubiquitously expressed in all tumors [28]. Here we observed a correlation between PDGFR-B expression and *NF2* loss in human meningiomas and their synergy in mouse meningiomagenesis.

Based on the evidence of PDGF-B and PDGFR-B expression in meningiomas, several pre-clinical and clinical trials have been performed using PDGFR. Meningioma cell lines demonstrated the efficacy of Sunitinib inhibiting cell proliferation and migration *in vitro* and that PDGFR was the prime target of the drug. Sunitinib reduced PDGFR-autophosphorylation even at the lowest concentration tested [29]. After disappointing results of a phase II trial of imatinib mesylate, a recent single arm phase II study on another Receptor Tyrosine Kinase (RTK) inhibitor targeting PDGFR and VEGFR, sunitinib malate, demonstrated partial activity in recurrent and progressive Grade II and III meningiomas, encouraging additional trials [30].

We found no difference in meningioma histological subtypes in PDGF-induced tumors compared to previous models, with a predominance of meningothelial, fibroblastic and transitional types [7, 9]. On the other hand, PDGF-induced tumors were mostly found at the convexity while meningiomas in the other mouse models were mostly found at the skull base [7, 9]. Interestingly, PDGF-B overexpression in PGDStv-a expressing cells also induced gliomas of various histological grades. This is likely due to PGDS expression in oligodendrocytes after commencement of myelination in rats [31–33], mice [34] and humans [8, 35] making it a marker of mature oligodendrocytes [36]. In this model, gliomas were more frequent than meningiomas in *PGDStv-a*;RCAS-PDGF-B mice. In other PDGF-driven glioma mouse models, the incidence of tumors was 33% at 3 months when targeting oligodendroglial progenitors using the CNP promoter (*Ctv-a* mouse, [19]), 50% at 3 months when targeting neural/glial stem cells with Nestin promoter (*Ntv-a* mouse, [37]) and 16% at 3 months when targeting astrocytes using GFAP promoter (*Gtv-a* mouse, [37]). The high incidence of gliomas in our model at 8 months suggests that other cell types in the CNS and/or developmental stages are sensitive to PDGF-B transformation and may serve as cell of origin for glioma.

Finally, we have confirmed the key role of *Cdkn2ab* loss in meningioma malignant transformation. While PDGF-B overexpression in addition to *Nf2* loss resulted in higher tumor frequency and grade II meningiomas, additional loss of *Cdkn2ab* also induced malignant grade III meningiomas.

In conclusion, we showed the pivotal role of PDGF-B in meningioma initiation and progression thus confirming it as a target for future clinical trials with selective inhibitors.

## MATERIALS AND METHODS

### Immunohistochemical characterization of PDGFR-B in human NF2- and NF2+ meningiomas

A Tissue Micro Array was built using meningiomas of all grades operated in the Neurosurgery department of Beaujon Hospital, Clichy, France. The local institutional review board approved this retrospective analysis. All patients or their parents provided informed consent. Sonic aspirator extracts were excluded from the study. Meningiomas were graded based on WHO 2000 criteria. Immunostainings for PDGFR-B (1:1, clone E29, DAKO) was performed for all cases. *NF2* gene characterization was performed as previously published [38], with additional NF2 protein Western Blot. Tumor material and NF2 status were available for 54 patients. There were 25 WHO Grade I, 26 WHO Grade II and 3 WHO Grade III meningiomas.

### Generation and genotyping of PGDStv-a transgenic mice

All animal experiments were performed in accordance with the local animal ethics committee.

Germline chimeras (*PGDStv-a*^floxGFPHygro/+^) were generated by injection of 10 mutant embryonic stem cells into C57BL/6 blastocysts, and crossed with FVB/N mice to produce outbred heterozygous offspring. The genotypes of all offspring were analyzed by polymerase chain reaction or Southern blot analysis on tail-tip DNA. To generate *PGDStv-a* mice, *PGDStv-a*^floxGFPHygro/+^ mice were crossed with EIIACre deletor transgenic mice [39]. In the deriving double transgenic offspring *Xba*I–*Nde*I digested tail DNA, deletion of the floxed GFP-Hygromicin cassette was confirmed by PCR. Mice carrying the *PGDStv-a* allele were subsequently crossed with FVB/N mice to segregate the mutant allele. Genotyping was carried out on DNA purified from tail biopsies after PCR reactions were performed using primers specific for *PGDStv-a* (sequence available upon request).

### *In vitro* infection and immunohistochemical staining of primary arachnoid cultures

Primary arachnoid cell cultures were established from adult *PGDStv-a*^−/+^ mice. The arachnoid tissue was surgically removed at the ventral surface of the brainstem after sacrifice and plated in Petri dishes after 1 h digestion by Collagenase. Arachnoid cells were then cultured in Dulbecco's modified Eagle's medium (DMEM; Gibco/Invitrogen, Carlsbad, CA, USA) containing 10% Fetal Calf Serum (FCS), Insulin and Epidermal Growth Factor (EGF). Cells were infected for 5 days for characterization and seeded on coverslips for immunohistochemistry. Infected cells were fixed in 4% PFA for 15 min and permeabilized with 0.1% Triton X. They were then incubated with primary and secondary antibodies. Cells were mounted in Vectashield (Vector Laboratories, Burlingame, CA, USA) containing DAPI. Stainings were visualized using a Zeiss (Oberkochen, Germany) microscope.

### Immunostaining of postnatal brain sections

After injection of newborn mice with DF-1 cells producing RCAS-eGFP retrovirus at PN1 and Adenovirus Ad*β-Gal* at PN3, pups were sacrificed and injected P7 heads injected at were fixed in 4% Formol at 4°C overnight and cryosectioned. Sections were incubated with primary antibody Pgds (1/1000; Santa Cruz, sc-14825), GFP (1/200; Abcam, catalog#ab290), diluted in blocking solution followed by secondary antibody. Slides were mounted Vectashield (Vector Laboratories, Burlingame, CA, USA) containing DAPI. Stainings were visualized using a Zeiss (Oberkochen, Germany) microscope.

### *In vivo* tumor induction

For *Nf2* and *Cdkn2ab* inactivation, we used *Nf2*^flox2/flox2^ and *p16*^Ink4a−/−^;*p15*^Ink4b−/−^;*p19*^Arf flox/flox^ (noted *Cdkn2ab*^−/−^ in this paper) mouse strains [9]. Neonatal mice were injected subdurally as previously described [7] with 3 to 4 μl of Adenovirus (Ad5CMV-Cre-*AdCre* or Ad*β-Gal*) (University of Iowa Gene Transfer Vector Core, Iowa City, IA, USA) at PN1 and then injected at PN3 with 4 μl of RCAS-producing DF-1 chicken fibroblasts (RCAS-PDGF-B, RCAS-GFP), which equals approximately 2.10^5^ cells, as previously described [19]. RCAS-PDGF-B-HA contains a 3-nt ‘ACC’ Kozak consensus sequence in front of PDGF-B and a C-terminal HA epitope tag [40]. DF-1 cells were cultured in DMEM with addition of 10% FCS (Gibco), 4 mM L-glutamine (Gibco) and 1% penicillin/streptomycin (Gibco). Mice were monitored every day and euthanized on any sign of illness or at predefined time points according to the control cohorts. Brains were then fixed, decalcified and embedded in paraffin as previously described [41].

### Immunohistochemical analysis of tumors

Immunohistochemical staining was performed on 6-mm paraffin sections. For antigen retrieval, after pressure boiling in antigen unmasking solution (Vector Labs, Burlingame, CA, USA) or 1 mM EDTA (pH 8.0) for 45 minutes, sections were incubated in 1% H2O2 in methanol for 30 min and blocked in 1.5% Normal Goat Serum followed by incubation with primary antibody. Biotinylated secondary goat anti-mouse, goat anti-rabbit, rabbit anti-goat or rabbit anti-rat antibodies were used at 1:200. Stainings were developed using Vectastain ABC system and DAB substrate kit (Vector Labs). For HA secondary antibody Alexa Flour 555 donkey anti-rabbit was used at 1:400 dilution and mounted in Immu-Mount (Shandon) containing DAPI.

### Proliferation assay

After 10 days of primary culture, mouse arachnoid cells were passaged in 6-well plates at a concentration of 1000 cells / mL. After 72 hours, cells were infected with AdCre at a concentration of 50 pfu per cell for 48 hours. Next, cells were infected with RCAS-PDGF-B and RCAS-GFP (GFP-vec) as described [19]. Infection was repeated daily for 5 consecutive days. Cells were then seeded at a concentration of 2.10^4^ cells/mL in triplicate, and counted 1, 4 and 7 days after plating, manually using blade KOVA (Hycor, IN, USA) and/or automatically using Cellometer (Nexcelomn Nexcelom Bioscience, MA, USA).

### Western blot

Cells were washed twice in ice-cold PBS, scraped in PBS and lysed in a buffer containing 50 mM Tris-HCl pH 7.4, 150 mM NaCl, 1% P40, 0.25% sodium deoxycholate, 1 mM EDTA, prepared from a 10X solution (RIPA Lysis Buffer, Millipore) to which was added a cocktail of protease inhibitors (Complete™ Protease inhibitor cocktail, Roche). The samples were incubated for 45 min on ice, then centrifuged for 20 min at 16,000 g at 4°C. The resulting supernatant was removed and the protein concentration of each extract was determined using a DC Protein Assay (Biorad, CA, USA). The proteins were separated by migration in a gel concentration and migration SDS-PAGE and transferred to a PVDF membrane (Immobilon-P Transfer, Millipore) at 4°C for 2 hours at 100 V, blocked using a solution of BSA (Bovine Serum Albumin from 96%, Sigma Aldrich) diluted to 5% in Tris-HCl pH 7.6, 0.1 M NaCl, 0.1% Tween (TBS-T), washed with TBS-Tween 0.1% and immunoblotted with the primary antibody monoclonal anti-PDGFR B (Cell Signaling, catalog#3169, clone#28E1) rabbit diluted by 1/1000 and the rabbit anti with Tag-HA (Santa Cruz, catalog#sc805, clone#Y-11) diluted by 1/1000. The secondary antibody used was anti-rabbit polyclonal IgG HRP (Santa Cruz, catalog#sc2004) diluted by 1/10000.

## SUPPLEMENTARY FIGURE


