# Supplementary figures and images for: PDGF activation in PGDS-positive arachnoid cells induces meningioma formation in mice promoting tumor progression in combination with Nf2 and Cdkn2ab loss
Source: Oncotarget. 2015 Sep 24;6(32):32713–22. doi: 10.18632/oncotarget.5296 (PMC4741724; doi:10.18632/oncotarget.5296)

## SUPPLEMENTARY FIGURE

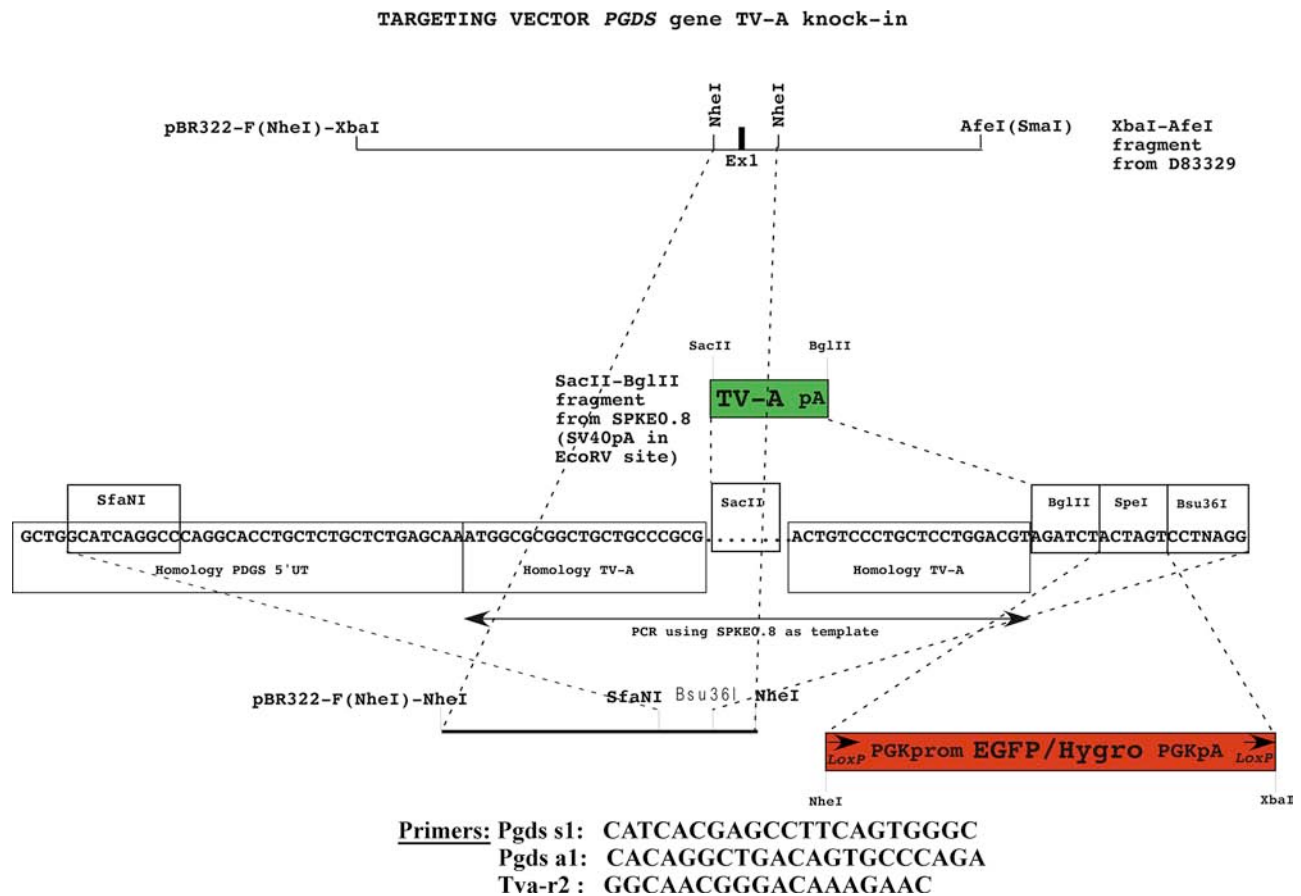Supplementary Figure S1: Construction of the PGDS<sup>tv-a</sup> allele.

Supplement: Supplementary file 1 [file oncotarget-06-32713-s001.pdf]
